# Contamination Event Detection with Multivariate Time-Series Data in Agricultural Water Monitoring †

**DOI:** 10.3390/s17122806

**Published:** 2017-12-04

**Authors:** Yingchi Mao, Hai Qi, Ping Ping, Xiaofang Li

**Affiliations:** 1 College of Computer and Information, Hohai University, Nanjing 211100, China; qihai@hhu.edu.cn (H.Q.); amazingapple@hhu.edu.cn (P.P.); 2 School of Computer Information & Engineering, Changzhou Institute of Technology, Changzhou 213032, China; lixf@czu.cn

**Keywords:** event detection, back propagation model, multivariate water quality parameters, time-series data, spatial-temporal model, connected dominating set, water supply network

## Abstract

Time series data of multiple water quality parameters are obtained from the water sensor networks deployed in the agricultural water supply network. The accurate and efficient detection and warning of contamination events to prevent pollution from spreading is one of the most important issues when pollution occurs. In order to comprehensively reduce the event detection deviation, a spatial–temporal-based event detection approach with multivariate time-series data for water quality monitoring (M-STED) was proposed. The M-STED approach includes three parts. The first part is that M-STED adopts a Rule **K** algorithm to select backbone nodes as the nodes in the CDS, and forward the sensed data of multiple water parameters. The second part is to determine the state of each backbone node with back propagation neural network models and the sequential Bayesian analysis in the current timestamp. The third part is to establish a spatial model with Bayesian networks to estimate the state of the backbones in the next timestamp and trace the “outlier” node to its neighborhoods to detect a contamination event. The experimental results indicate that the average detection rate is more than 80% with M-STED and the false detection rate is lower than 9%, respectively. The M-STED approach can improve the rate of detection by about 40% and reduce the false alarm rate by about 45%, compared with the event detection with a single water parameter algorithm, S-STED. Moreover, the proposed M-STED can exhibit better performance in terms of detection delay and scalability.

## 1. Introduction

Water is an important resource of the ecological environment in the agricultural industry. Polluted water can have directly negative impact on the agricultural irrigation and result in the low quality and output of the crops. When a large-scale water contamination event occurs, it is one of the most important issues to detect and warn contamination events and prevent pollution from spreading. One effective way is to deploy a large-scale number of water quality sensors to monitor generic water quality parameters and detect contamination events [1]. At present, online water quality sensors are deployed in the waters networks (WSNs), which can measure many contaminants and provide an early indicator of possible pollution [2,3]. Contamination event detection approaches focus on identifying abnormal system behavior. Accurate detection should consider the response strategies, operation optimization, and overall system efficiency [4]. It is an important to establish how to detect contamination events in real-time and in an accurate way with the multivariate time series of water quality parameters in a WSN. The main water quality parameters are free chlorine, electrical conductivity (EC), pH, temperature, total organic carbon (TOC), and turbidity [5]. Almost all existing contamination event detection approaches are based on only a single water quality parameter, which results in high false negative rates and low false positive rates [6]. It is difficult to detect a potential contamination event in the WSNs due to the number of parameters involved [7].

In real application, many redundant sensor nodes are deployed in the water supply networks to improve the robustness of the monitoring systems. On the other hand, due to large-scale sensor node deployment, the amount of data from dissimilar sensors has increased tremendously. To reduce the computational cost of event detection and improve the response time, sensor node selection needs to cut down the amount of data for the spatio-temporal correlation analysis. To avoid forwarding and collecting the sensed data from all of the sensors in the WSNs, Connected Dominating Set (CDS) [8,9] is introduced to select the backbone nodes and construct an effective connected structure in a WSN to reduce the amount of transmitted data and improve the response speed for contamination event detection. In this paper, a distributed and effective algorithm to construct CDS called Rule **K** [9] is adopted to select backbone nodes and forward the sensed data of water quality.

Meanwhile, real-time event detection (e.g., water pollution incidences) in the WSNs should consider both spatial and temporal correlation and dynamically record the propagation path of the contamination event. This paper focuses on real-time contamination event detection with a spatio-temporal correlation model in a water supply network to achieve high accuracy and low false alarm rates. Therefore, a spatial–temporal-based event detection approach with multivariate time-series data for water quality monitoring (M-STED) was proposed. The M-STED approach includes three parts. The first part is that M-STED adopts Rule **K** algorithm to select backbone nodes as the nodes in the CDS, and forward the sensed data of multiple water parameters. The second part is to determine the state of each backbone node with back propagation neural network (BP) models and the sequential Bayesian analysis in the current timestamp. The third part is to establish the spatial model with Bayesian networks to estimate the state of the backbones in the next timestamp and trace the “outlier” node to its neighborhoods to detect a contamination event.

The contributions of this work are as follows.

(1) A connected dominated set (CDS) approach is introduced to select backbones from a large number of sensors, which can cut down the amount of data for the spatio-temporal correlation analysis.
(2) A data-driven event detection algorithm is developed, which relies on multivariate water quality time series with Bayesian sequential analysis and BP model from a WSN. Multiple parameters of water quality are fused to provide a unified decision support about a contamination event.
(3) Bayesian network (BN) is used to model spatial dependency.

The experimental results indicate that the proposed M-STED approach can achieve 90% accuracy with BP model and improve the rate of detection by about 40% and reduce the false alarm rate by about 45%, compared to temporal abnormal event detection of univariate spatial-temporal event detection algorithm (S-STED). Furthermore, we found that M-STED can achieve better performance in terms of response time and scalability, compared to the simple threshold algorithm and the BN-only algorithm.

The rest of this paper is organized as follows. Related work is discussed in Section 2. Section 3 presents the overall water supply network model with multivariate time-series of water quality parameters. The CDS-based node selection strategy is proposed in Section 4. In Section 5, the temporal event detection with multivariate time-series data algorithm for a single node is proposed. Section 6 presents the spatial correlation model for event detection. The performance evaluation is given in Section 7. Section 8 is conclusions.

## 2. Related Work

The contamination event detection approaches based on water quality measurements consist of two phases. The first phase is to set up the prediction model with the historical data as the training dataset. The second one is to determine the water quality by comparing the predicted values with the measurements. Various approaches have been proposed for addressing the problem of contamination detection, using single or multiple-type measurements which are analyzed separately or in combination, from one or more locations in the network, using model-based or model-free approaches.

In the threshold-based approaches, the threshold values are set through statistical models. Simplicity is the main advantage since raw data can be directly processed. The abnormal event detection with two thresholds were adopted in [10]. However, the threshold-based approaches cannot obtain the spatio-temporal feature of water quality data, which results in low accuracy and high false positives of event detection. In the pattern matching approaches, the pattern is established and verified with water quality sensor readings to infer the contamination event [11]. Byer and Carlson assumed that the water quality parameters obey a Gaussian distribution. One statistical model was established to detect contamination events [12]. The statistical model detection methods cannot be used in the applications.

Learning-based methods can make inference of the possibility of contamination events based on the temporal and spatial correlation of water quality measurements [4,13,14]. It was promising to make full use of the spatial and temporal correlation to detect contamination events. Markov random fields (MRFs), Bayesian network (BN), dynamical BN, and SVM are common models in the WSNs with the high density. MRFs were adopted to model spatial context and stochastic interaction among observable quantities [15]. BN is considered as a means for unsupervised learning and anomaly detection in gas monitoring sensor networks for underground coal mines [13]. Using a single water parameter-free chlorine, radial basis function (RBF) neural network and wavelet analysis were applied to determine the contamination event occurrence with on-line data of water quality [16]. Perelman proposed a BN-based contamination event detection method to determine the event occurrence through estimating the possible locations of potential contaminants in WSNs [17]. One improved water-contamination events detection based on D-S theory was proposed to predict water quality parameters with on-line water quality sensors [18]. The contamination event detection algorithm based on principal component analysis (PCA) has been presented [19]. The PCA was applied to the normalized measurement. Then, the alarm index and the threshold of the alarm were obtained. In addition, a spatio-temporal model was adopted to detection the contamination events with water sensor networks [20].

A multiple type measurement approach at a single location was proposed in [12], where each parameter was compared to its three bounds. Control charts and Kalman filters have also been proposed in [21]. When multiple types of sensors are available, these can be used to compute distance metrics to detect contamination [22]. The probability of contamination events could be computed and compared to an adaptive threshold by utilizing a sequential Bayesian rule [23]. The estimation error of event detection was computed with respect to the measurements from a moving window [24]. Moreover, the US Environmental Protection Agency provides the event detection tool-CANARY [25,26] for time series analysis of multiple water quality parameters. Fluctuations in water distribution networks may cause significant variability, a Bayesian belief network was presented to infer the probability of contamination [27].

Based on the above analysis, most approaches for contamination event detection have been discussed via using single type water quality parameters. There are multiple components to indicate water quality in a WSN, such as free chlorine, EC, pH, temperature, TOC, and turbidity. Unfortunately, a single parameter of water quality cannot reflect the real water quality in a WSN. When a contamination event occurred, the observable values of multiple water quality parameters changed in a significant way. The contamination event detection methods based on single parameter may result in low detection accuracy and high false alarms. To improve the detection accuracy and reduce the false alarms, multiple parameters of water quality should be considered to make a decision with data fusion. Liu et al. [28] proposed a method for real-time contamination detection using multiple conventional water quality sensors. Eight sensors were used in the case study. Furthermore, they extended their work by determining how the number of sensors influences the detection performance and identifying the optimal combination of sensor deployment [29]. Unfortunately, the development of multi-parameter water quality models and their calibration is challenging due to the large amount of information and number of parameters involved. A large amount of data should be transmitted and processed, which results in the great overhead for event detections. Thus, their application in the context of contamination event detection is complicated. On the other hand, changes in sensor readings caused by one contamination event usually exhibit a strong spatio-temporal correlation. The spatial and temporal correlation is critical to improve the accuracy of event detection. In this paper, the proposed M-STED utilizes a back propagation neural networks model to estimate the relationships between water quality parameters in a WSN. The proposed M-STED can detect potential contamination events for temporary analysis of multivariate water quality time series with Bayesian sequential analysis.

## 3. Water Supply Network Model

**Figure 1:**
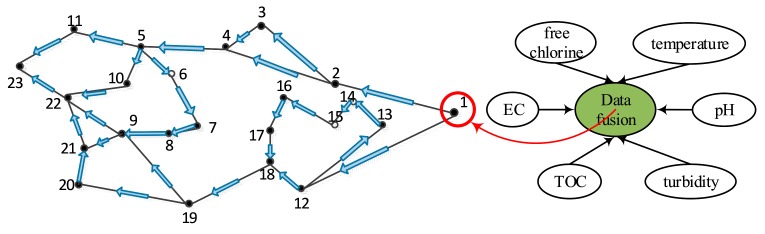
The topology of a water supply network.

To clearly illustrate the event detection with spatio-temporal correlation in water sensor networks, the structure of a water supply network is shown in Figure 1. All of water quality sensors are deployed in the water supply network and each sensor can communicate with others via single-hop communication. The topology of the water supply network can be represented as a graph G=(V,E), where V is the set of water quality sensor nodes. Each directed edge (u,v)∈E in the graph G can be represented as the single-hop transmission link. In the water supply network, a set of sensors is deployed to monitor water quality. Each sensor node has a unique ID, from 1 to n. Sensors can sense a variety of attributed, such as free chlorine, electrical conductivity (EC), pH, temperature, total organic carbon (TOC), and turbidity.

Before contamination event detection, the spatio-temporal correlation is modeled based on normal historical data. For a large water supply network, it is difficult to simultaneously model spatial and temporal relationships due to the complexity of the data. In this paper, spatial and temporal relationships are separately modeled. BP models and the sequential Bayesian analysis are used to detect the outlier nodes for each water quality sensor node with multivariate time series of six water quality parameters. The conditional probability of BN is used to represent the spatial relationship. $S_{i}^{t}$ is denoted as the observed value from sensor $S_{i}$ at the time interval t. Once n−tuple $\left(S_{1}^{t},S_{2}^{t},\ldots ,S_{m}^{t}\right)$ can be used as one of the training data cases for learning the parameters of the BN. The maximum likelihood parameter estimation algorithm is adopted to compute the parameters of the BN. If the states of corresponding sensor nodes deviate from the learned spatial relationship, we consider that a contamination event has occurred.

In this paper, the proposed M-STED approach keeps monitoring streams from the backbone nodes at every timestamp and traces from one outlier to its neighborhood to find a contamination event. First, the water supply network keeps monitoring data from the sensor nodes in the CDS at every timestamp, and then the selected nodes analyze whether a temporal outlier appears with BP models and sequential Bayesian analysis for each node in the CDS. If it does not, the system waits to monitor the data streams from the backbone nodes in the next timestamp. Otherwise, a candidate CDS set is generated for each “outlier” node and its children nodes of each “outlier”. Then, in the next timestamp, the monitoring scope will be expanded, including the backbone nodes and the nodes in the candidate sets. Once a node in a candidate set is detected as a spatio-temporal outlier node, the children nodes of the outlier node will be added to the candidate set. When the number of spatio-temporal outliers is equal to or greater than the threshold θ, we consider that a contamination event has happened and the corresponding warning is broadcast.

## 4. Backbone Node selection

Thousands of water quality sensors are deployed in a water supply network to sense, transmit, and forward the massive sensed data, which brings heavy burdens to further contamination event detection in the context of real-time analysis. To avoid forwarding and collecting all of the sensed data from all sensors in the WSNs, optimal sensor node selection is needed to reduce the amount of transmitted data and improve the response time for event detection. CDS is one of the optimal node selection strategies from Graph Theory [8].

A dominating set (DS) of an undirected graph G=(V,E) is a subset of $V^{\prime }$ of the vertex set V such that every vertex in $V-V^{\prime }$ is adjacent to a vertex in $V^{\prime }$. A dominating set $V^{\prime }$ is connected if for any two vertices u and v, a path $\left(u,v_{1}\right)$, $\left(v_{1},v_{2}\right)$, …, $\left(v_{n},v\right)$, $\left(v_{i}\in V^{\prime },1\leq i\leq n\right)$ in E exists. Rule **K** algorithm was proposed in our previous work [9], which is an energy efficient and distributed algorithm for CDS selection. Through the selection of the appropriate communication range, a set of CDS can be constructed to meet the requirements of network connectivity and coverage. In each round of selection, the sensors adjust the appropriate communication range and select the nodes as nodes in the CDS. The sensors in the CDS as the backbones execute the tasks of monitoring and data forwarding. After the collected data from the CDS are analyzed, the system can detect the contamination event in the context of real-time analysis. The backbone nodes can keep monitoring several streams from backbone nodes instead of all streams, therefore, the CDS-based node selection strategy can reduce the amount of transmitted data and improve the response time for real-time event detection. In this paper, Rule **K** algorithm [9] is adopted to select the backbone nodes and construct an effective connected structure in a WSN.

Rule **K** algorithm consists of two parts: marking process and pruning rule.

In the marking process, all of the water quality sensor nodes are non-dominated nodes. Each node u exchanges its neighbor’s information in its communication radius $R_{u}$ with all its neighbors. According to two neighbors’ information, node u judges whether it can directly communicate with its neighbor nodes v and w or not. That is $d(u,v)\leq R_{u}$, $d(u,w)\leq R_{u}$, while $d(w,v)>\min\{R_{w},R_{v}\}$. If two nodes v and w can directly communicate, node u can be as a candidate dominant node and participate in the construction CDS. Otherwise, node u is controlled by other nodes and is not in the CDS.

In the pruning process, it should reduce the size of dominant node set because the number of candidate dominant node selected via marking process is excessive. The basic idea of pruning rules is that if the k neighbor nodes of candidate dominant node u can make communication with other nodes, then node u can be deleted from the CDS. The CDS-based node selection strategy can reduce the amount of transmitted data and improve the response time of the event detection.

## 5. Temporal Event Detections with Multivariate Time Series in a Single Node

### 5.1. Main Idea

**Figure 2:**
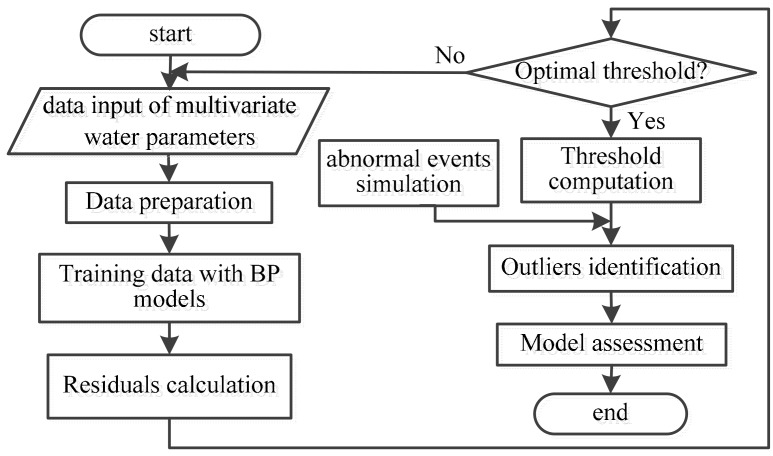
Procedure in the off-line phase.

**Figure 3:**
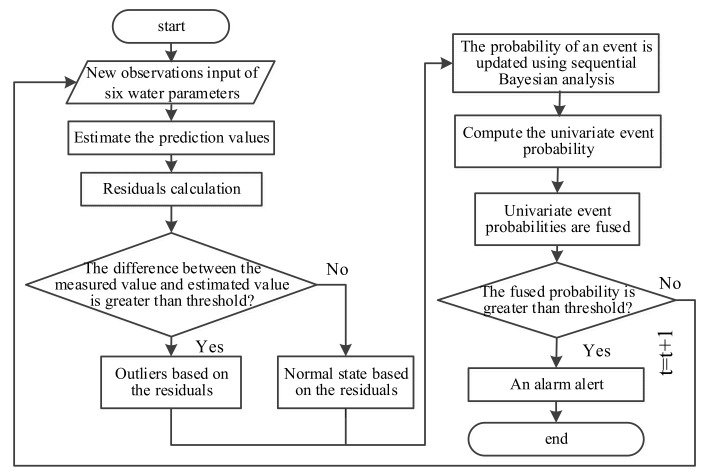
Procedure in the on-line phase.

The temporal outlier detection approach consists of two phases: an off-line phase and an on-line phase. At first, the system is in the off-line phase. During the off-line phase, one data-driven model—BP neural network is established. The historical data is trained for temporary analysis of multivariate water quality time series. If the obtained model has low residuals, the system enters into the on-line phase manually. In the on-line phase, Bayesian sequential analysis and BP model are adopted to compute the new measured parameter’s value of each water parameter. Six parameters of water quality are fused to provide a unified decision support about a contamination event. After a period of time, with the increase of the measured values, it needs to retrain the historical data to refine the assessment model. A summary of the overall temporal outlier detection framework is presented in Figure 2 and Figure 3.

Off-line phase: First, six BP neural networks were established and trained to simulate the six water quality parameters (free chlorine, EC, pH, temperature, TOC, turbidity), respectively. Then, the residuals are calculated for each new measured value of each quality parameter as the total error. The possible outliers can be identified based on the residuals. The next step is to evaluate the sensitivity of the BP model and assess its accuracy.

The on-line phase includes four main steps for each incoming measured value: (1) data-driven model training—the relationships between water quality parameters are examined using BP models; (2) error threshold setting—calculated residuals are classified as normal or abnormal values using thresholds for each water quality parameter; (3) identification of events—the probability of an event is updated using sequential Bayesian analysis based on the residual classification; and (4) information fusion—univariate event probabilities are fused to provide a unified multivariate event probability reflecting the likelihood of an event based on all water quality parameters. Each water quality parameter is assigned a weight reflecting its influence on the fusion decision. Steps (1) and (2) are initially trained using available data collected from the WSN. Steps (1) to (4) are then repeatedly executed in real-time for each new incoming measured value.

### 5.2. Water Parameters Simulation Based on BP Model

BP model is adopted to estimate the relationships between six water quality parameters through nonlinear, weighted functions during normal operation. The development of BP model does not require any knowledge of the physical and chemical laws affecting water quality parameters. Thus, BP model can be available to model the multivariate reactions in the WSN.

In the water supply networks, contamination events heavily rely on multiple water quality factors, which results in the difficulties to pre-specify the events. In the case of free chlorine, BP model is just established with the historical data from single water parameter—free chlorine to estimate the predicted value. When there is a small difference between the measured and estimated free chlorine values, it has obvious great deviations between the measured values of other water parameters and their normal states. The reason is that the measured values of free chlorine exhibit great deviations from the normal operation conditions due to the interplay among some of water quality parameters. The prediction model based on a single water quality parameter would obtain a lower accuracy of estimated values. To improve the accuracy of the prediction model, BP neural networks are adopted and trained for modeling the relationships between multiple variate water quality parameters in WSNs. One of the most important components in the BP model are multi-layer perceptrons (MLPs). MLPs are represented by an input layer, a hidden layer, and an output layer and correlate input variables to output variables depending on the nonlinear, weighted, parameterized functions. The BP model is shown in Equation (1):

(1) $$f_{k}(x;w)=\varphi_{0}(w_{0}+\sum_{j}w_{jk}\varphi (w_{0j}+\sum_{i}w_{ij}x_{i}))$$

where $w_{jk}$, $w_{ij}$ are weights, $w_{0}$, $w_{0j}$ are biases, φ and $\varphi_{0}$ are activation function and output function, respectively, $x_{i}$ is one of water quality parameters, and $f_{k}(x;w)$ is used to estimate the target value y. Data is passed from the input layer with p inputs $x=(x_{1},\ldots x_{p})$ to hidden layer having m neurons. Each neuron in the hidden layer receives the summed weighted outputs of the preceding layer. The output layer with k targets $y=(y_{1},\ldots ,y_{k})$ receives the summed weighted outputs of the preceding layer again. The final output is the function $f_{k}(x;w)$.

To avoid the interplay among multiple water quality parameters, for each target water quality parameter, the corresponding BP model is established with the input measured time series of all predictive parameters and lagged target parameter, as formulated in Equation (2).

(2) $${\hat{x}}_{i}(t)=f(x_{1}(t),\ldots ,x_{i-1}(t),x_{i}(t-1),x_{i+1}(t),\ldots ,x_{n}(t))$$

where $x_{i}(t)$ and ${\hat{x}}_{i}(t)$ are the measured and estimated water parameters at time t respectively, n n is the number of water quality parameters, and f(⋅) is a function defined by the BP model, as in Equation (1).

**Figure 4:**
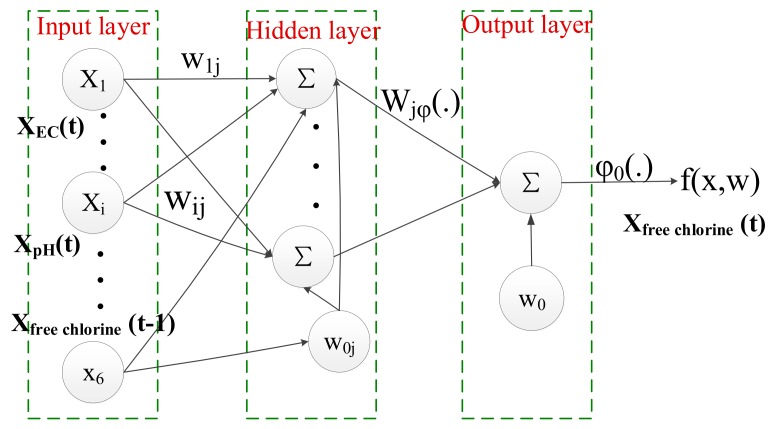
BP (Back Propagation) neural network structure of free chlorine.

Water quality parameters include free chlorine, electrical conductivity (EC), pH, temperature, total organic carbon (TOC), and turbidity. Six BP models can be created and trained. One of each water quality parameter $x_{i}(t),\;i=1,2,\ldots ,6$ with six water parameters, respectively, to predict the target water quality parameters and the relationships between them. A BP model consists of the size of the BP neural network, its inputs, and a training algorithm and testing the model with measured values. According to Equation (2), one model was constructed for each target water quality parameter with the input vector containing measured time series of all predictive parameters at the same time as lagged target parameters. For example, as shown in Figure 4, the BP model for estimating free chlorine takes the following inputs:

(3) $${\hat{x}}_{free\_chlorine}(t)=f(x_{EC}(t),x_{pH}(t),x_{temperature}(t),x_{TOC}(t),x_{turbidity}(t),x_{free\_chlorine}(t-1))$$

The accuracy of BP model can be evaluated through mean error, standard deviation (STD), and correlation ($R^{2}$) between the measured values and predicted values of water quality parameters.

### 5.3. Residual Estimation and Classification

The established BP models through training the historical data can be utilized to predict the values of water quality parameters. The next step is to calculate the residuals as the difference between the predicted and measured values of parameters and then to classify the residuals. Residuals can be calculated as shown in Equation (4), represented as time series.

(4) $$ER_{i}(t)=x_{i}(t)-f(\cdot )=x_{i}(t)-{\hat{x}}_{i}(t)$$

where $x_{i}(t)$, ${\hat{x}}_{i}(t)$, and $ER_{i}(t)$ represent the measured value, the estimated values, and the estimated residual for parameter i at the time t, respectively, f(⋅) is defined by Equation (1).

To distinguish the normal operation of a water supply network from the contamination events, the estimated residuals $ER_{i}(t)$ can be classified into two types: normal and outlier. For each BP model, the estimated residuals are bounded within the upper and lower limits. If the measured values exceeding the threshold are considered to represent outliers. During the training phase, the experimental results indicate that the majority of residual within the bound [96%, 99%] can be acceptable. If the residuals cannot reside within the upper and lower limits, the residual should be considered as outlier.

### 5.4. Sequential Bayesian Updating

In this stage, the probability of a contamination event is updated using sequential Bayesian analysis for each incoming value. In the sequential Bayesian analysis, the number of incoming values is unknown in advance. In fact, the measured values come in sequence and a decision is required to be made about the current state. The performance of the BP model is measured through a confusion matrix during the training [30]. The confusion matrix represents the model’s classification of all measured values to one of four classes: true positive (*TP*), the residual is classified as an outlier during an actual event; false oositive (*FP*), the residual is classified as an outlier during normal operation condition; true negative (*TN*), the residual is classified as reasonable model error during normal operation; false negative (*FN*), the residual is classified as a reasonable model error during a contamination event, as shown in Table 1.

Additional common metrics, two others can be derived from the confusion matrix as described in Equation (5).

(5) $$RD=\frac{TP}{TP+FN}\times 100\%,FAR=\frac{FP}{TN+FP}\times 100\%$$

where RD represents the ratio of the number of the detected contamination events to the number of the actual contamination events, and FAR is the ratio of the number of the detected false alarms to the total number of the determined contamination events.

To determine the possibility of a contamination event occurrence from previously classified states (normal or outlier), the probability of a contamination event is updated based on the classification of the residuals. The state of an event has two states: normal and outlier, as shown in Equation (6). In the sequential Bayesian analysis for each new incoming data, the number of measured values is not known in advance. The measured values come in sequence, and a decision is made based on the current state.

(6) $$P(E_{t})=\left\{ \begin{array}{l} P(E_{t}\;|\;O_{t})\;\mathrm{if}\;{\mathrm{Residual}}_{t}\;\mathrm{is}\;\mathrm{Outlier}\\ P(E_{t}\;|\;\bar{O_{t}})\;\mathrm{if}\;{\mathrm{Residual}}_{t}\;\mathrm{is}\;\mathrm{Normal} \end{array}\right.$$

At first, the probability of a contamination event is assumed to be rare. With each new incoming measured value, the probability of an event is updated with sequential Bayesian rule, as shown in Equation (7).

(7) $$\begin{array}{c} P(E_{t}\;|\;O_{t})=\frac{P(O|E)\times P(E_{t-1})}{P(O\;|\;E)\times P(E_{t-1})+P(O\;|\;\bar{E})\times P({\bar{E}}_{t-1})}=\frac{RD\times P(E_{t-1})}{RD\times P(E_{t-1})+FAR\times (1-P(E_{t-1}))}\\ P(E_{t}\;|\;{\bar{O}}_{t})=\frac{P(\bar{O}\;|\;E)\times P(E_{t-1})}{P(\bar{O}\;|\;E)\times P(E_{t-1})+P(\bar{O}\;|\;\bar{E})\times P({\bar{E}}_{t-1})}=\frac{\left(1-RD)\times P(E_{t-1}\right)}{\left(1-RD)\times P(E_{t-1})+(1-FAR)\times (1-P(E_{t-1})\right)} \end{array}$$

where $P(E_{t})$ is the probability of an event at time t, $O_{t}$ and ${\bar{O}}_{t}$ are “Outlier” and “Normal” states at time t, respectively. $P(E_{t}|O_{t})$ is the conditional probability of a contamination event when the residual is classified as an outlier. $P(E_{t}|{\bar{O}}_{t})$ is the conditional probability of a contamination event when the residual is classified as a normal state.

From Equation (7), each event probability is updated through the sequential Bayesian rule depending on the new measured value and on the RD and FAR. The posterior probability is updated for each water quality parameter independently resulting in univariate event probabilities. The probability of events designates the likelihood of a contamination event based solely on the target parameter. If the probability exceeds the pre-defined threshold value, it is considered that a contamination event has occurred.

In the application of contamination event detection, the initial probability of an event is set to 10^−5^, and the default threshold probability for a contaminating event is set to $P_{threshold}=0.7$. Real data are available from CANARY database. The data covers four months with five-minute intervals of operations. Table 2 shows the average results of multiple runs for ten contamination events. In the contamination events simulation, the update probability of each water quality parameter can be found in Table 2. In the simulation, if the updated probability of single water parameter above 9.4 × 10^−1^ indicates an event. Alarm (1) represents at least one water quality parameter raised an alarm. Alarm (2) shows that an alarm is raised if at least two water quality parameters or more are fused. In this case, only one event is undetected with one false alarm. As shown in Table 2, one of the six water quality parameters identified a contamination event at 8:20, and three of the six parameters raised an alarm at 9:00. Moreover, during the contamination event spreading phase, the updated probabilities of a single water parameter gradually increased and more alarms are declared from 8:20 to 16:20. When the event ends, the pollution disappears slowly and the corresponding probability decreases.

### 5.5. Multivariate Fuse Decision

To improve the model’s estimation of a temporal event and to lower the number of false alarms, a process of multivariate fusion decision is invoked. This process ensures that only when a predetermined number of parameters indicates an event, an alarm is raised. This process reduces the number of false alarms and improves the alarms’ reliability. The univariate event probabilities are fused to compute the multivariate event probability reflecting the likelihood of a contamination event based on all measured water parameters.

**Figure 5:**
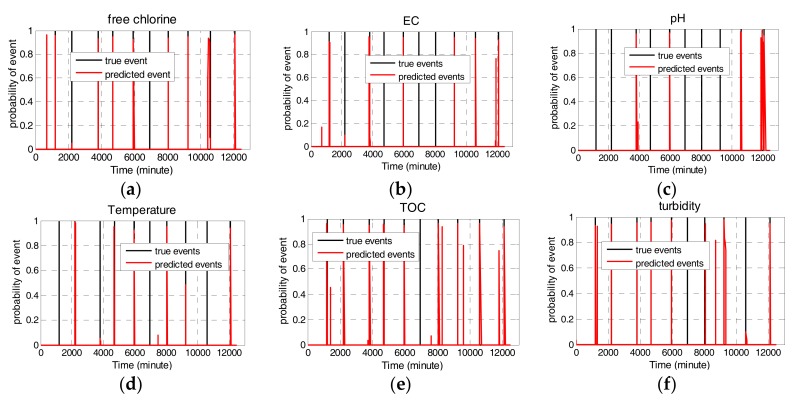
Event probability of single water quality parameter. (**a**) free Chlorine; (**b**) EC; (**c**) pH; (**d**) Temperature; (**e**) TOC; (**f**) turbidity.

Figure 5 illustrates the univariate event probabilities for each water quality parameter during the simulated ten contamination events. In the simulations, the threshold probability for a contamination event is set to $P_{threshold}=0.7$. If the updated probability of one water parameter is above 0.7, the corresponding event for single parameter occurs. As shown in Figure 5, free chlorine can detect eight out of ten events with three false alarms. EC can detect six out of ten events with one false alarm. pH can detect five out of ten events with one false alarm. Temperature detects five out of ten events without false alarm. TOC detects nine out of two events with three false alarms. Turbidity can detect eight out of ten events with two false alarms.

**Figure 6:**
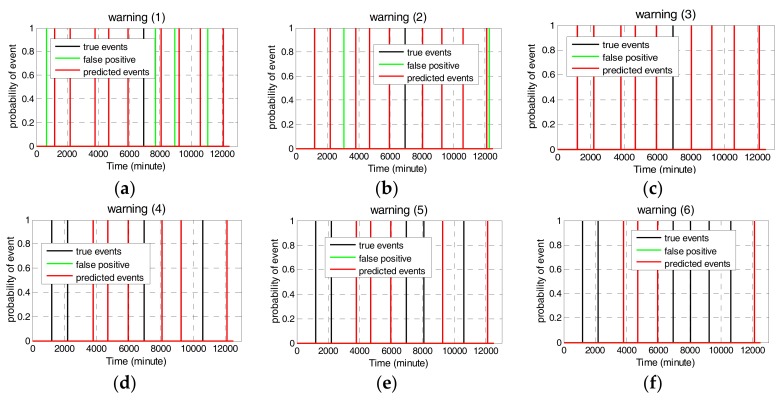
Event probability of multiple water quality parameters.

Since water quality parameters differ in their predictive ability of contamination events, weights reflecting their influence on the synchronized decision should be allocated. In this paper, uniform weights were given to reflect no prior information. Figure 6 depicts the multivariate fused alarms with six water parameters. In Figure 6, warning (1) represents that a contamination event is detected when the probability from one water parameter is above the threshold. In this case, almost contamination events can be detected, however, it also results in high false alarms. As shown in Figure 6, nine out of ten events can be detected with four false alarms. Warning (2) represents that a contamination event is detected when the probabilities from two or more parameters are greater than the threshold. In this case, only one true event cannot be detected with two false alarms. When the probabilities from three parameters are fused to indicate an event, there are no false alarms. This reflects the better tradeoff between the detection accuracy and the false alarm rate. Therefore, when the updated probabilities of three or more water parameters through the sequential Bayesian analysis are greater than the threshold 0.7, the M-STED event detection method can determine a temporal outlier occurrence after multiple fusion decisions in the water supply network.

## 6. Spatial Event Detection in the CDS

**Figure 7:**
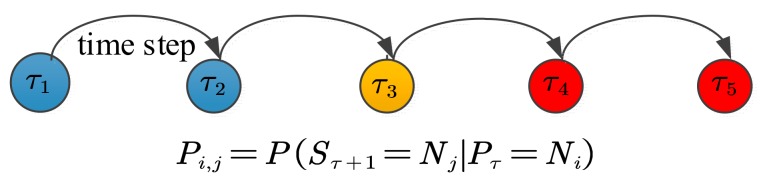
State transitions of the fused values of six water quality parameters at the moment $\tau_{1}$

In Section 5, single water quality sensor node in the water supply network can determine whether it is “outlier” or “normal” with multivariate time series of six water quality parameters. Unfortunately, it cannot obtain an accurate judgement for contamination event detection without considering the spatial correlations among the sensor nodes. After the determination the state of a single node, it should establish a spatial correlation model to further detect the contamination events based on the spatial relationship among the “outlier” sensor nodes. A BN is used to model causal and spatial relationship between the sensor stream and its neighbor sensor streams. As illustrated in Figure 7, a causal relationship exists between the upstream nodes and the downstream nodes, so the structure of BN is the same as the topological structure of water supply network.

.

Assume that there are three sensor nodes $S_{1}$, $S_{2}$ and $S_{3}$ to measure the multivariate water quality, with values varying from 0.0 to 1.0. There is a total of two states. Based on the experts’ experiences, if the values are in [0.0, 0.5), the state of sensor node is denoted as $state_{1}$. While the values are in [0.5, 1.0], the state is denoted as $state_{2}$. During the training phase, it can compute the parameters of BN by adopting the maximum likelihood parameter estimation algorithm.

After the training phase, the conditional probability table of each sensor can be learned. The temporal event probabilities of sensor $S_{1}$ are shown in Table 3. The conditional probabilities of sensor $S_{2}$ and $S_{3}$ are shown in Table 4. From Table 3, the probability of two states of sensor $S_{1}$ are 0.2 and 0.8, respectively. In Table 4, if the states of sensor $S_{1}$ is $state_{1}$, the state of sensor $S_{2}$ is $state_{1}$, then the conditional probability is 0.8. That is to say, $P(S_{2}=state_{1}|S_{1}=state_{1})=0.8$. During the reasoning phase, if the probability of sensor $S_{1}$ at a certain interval time is 0.24, the state of sensor $S_{2}$ will more likely at $state_{2}$ and the state of sensor $S_{3}$ will at $state_{1}$, according to the given conditional probability table. If the real value of sensor nodes $S_{2}$ and $S_{3}$ is not within that state, it can be considered that the sensed value deviates from the spatial relationship. It can be considered that a contamination event occurred.

The contamination event detection approach, M-STED, based on the spatio-temporal correlation model is as follows:

(1) The nodes in the CDS collect the sensed data. If temporal abnormal events occur, the nodes individually go through the BP model and sequential Bayesian updating with multivaries time series of six water quality parameters for a period of time τ. If the temporal event probability of one backbone node is greater than the threshold, it can consider that backbone node as in the “outlier” state.
(2) If the temporal event occurs in one of the backbone nodes, a candidate set is constructed with each backbone node in the “outlier” state, and its corresponding children nodes. If there is no temporal event in these backbone nodes, these nodes just wait for sensing and will be checked in the next time period.
(3) The reasoning ability of BN can be used to estimate the expected state of the backbone nodes $S_{i}$ in the “Outlier” state. Each backbone node compares the expected state with the observed value $S_{i}^{t}$ to determine whether the value of observed data deviates from the spatial relationship.
(4) If the observed value $S_{i}^{t}$ is greatly different from the expected value, it can be considered as a spatial deviation. The number of nodes in the “outlier” state increases by one.
(5) In the following timestamp, the scope of monitoring is expanded, including the backbone nodes and their candidate nodes. They continue to detect whether there is a temporal and spatial deviation.
(6) If the number of nodes in the “outlier” state in the CDS is equal to or greater than the threshold θ, we consider that a contamination event occurred and the warning should be broadcast.

## 7. Performance Evaluation

### 7.1. Experimental Setup

The methodology is tested on real data from CANARY [27]. The data spans over four months with five-minute intervals. The data consists of six online multivariate water quality parameters: free chlorine (mg/L), EC (mS/cm), pH, temperature (C), TOC (ppb), and turbidity (NTU). In the experiments, the data set is divided into two subsets, 67% for training and 33% for testing. The training subset is used to establish the BP neural network model with data-driven model, and the test subset is used to evaluate the accuracy of BP neural model.

**Figure 8:**
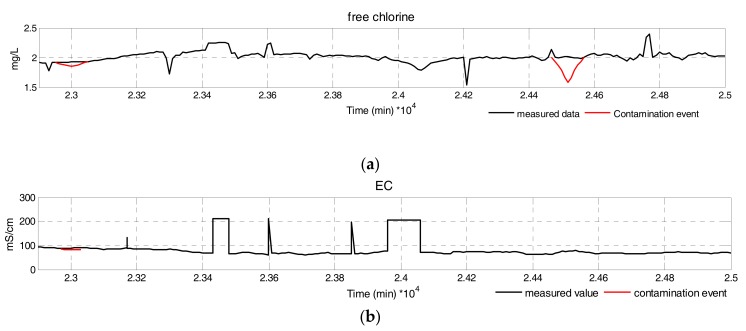

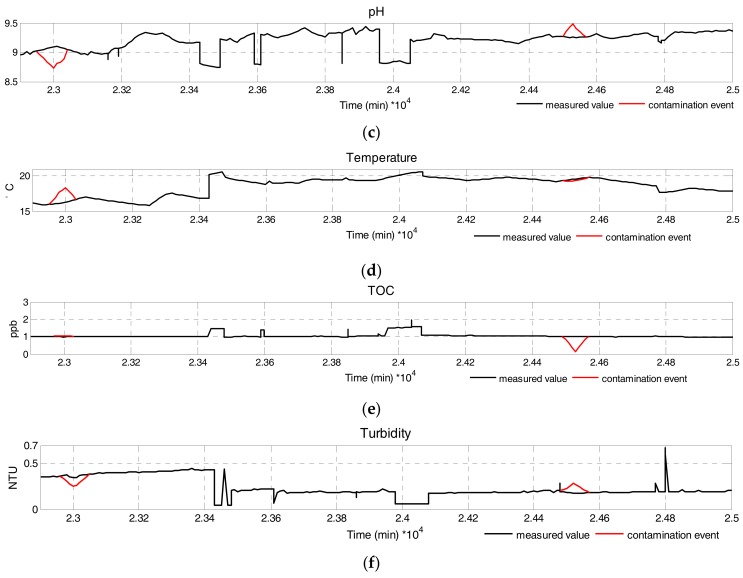
Time series of multivariate water quality parameters. (**a**) Free Chlorine; (**b**) EC; (**c**) pH; (**d**) Temperature; (**e**) TOC; (**f**) turbidity.

**Event Simulation:** Contamination events in the water supply networks heavily depend on environmental factors, which makes it harder to detect contamination events. To cope with the above difficulty, contamination events are superimposed on normal patterns, characterized by the magnitude, direction, and duration [31], which can reflect water parameter variation caused by contamination events. Contamination events were assumed to be Gaussian in distribution [32]. Figure 8 shows a partial time series plot of six water quality parameters during normal operation and randomly simulated events with real data from CANARY [27].

The experiments include three parts: (1) BP model assessment. The historical training subset of each water parameter was exploited to establish, train, and evaluate the BP model through mean square error (MSE), Correlation coefficient ($R^{2}$). The testing subset is used to simulate real-time contamination events and to test performance of the proposed M-STED approach; (2) Due to considering multivariate water quality parameters, several metrics, such as receiver operating characteristic (ROC) curve, rate of detection (RD), and false alarm rate (FAR), are used to evaluate the performance of the M-STED algorithm in terms of the accuracy, compared with the S-STED algorithm depending on a single water parameter; (3) Efficiency. The efficiency performance can be evaluated in terms of delay and scalability, compared with the simple threshold algorithm [10], Bayesian network-based algorithm [16]. The threshold-based approach is the basic one for the event detection in water supply networks. It has the lowest communication complexity. The BN-only approach only considers the spatial relationship among the sensor nodes without temporal relationship. The average delay is the average time from event occurrence to the event being detected. The shorter the average delay, the higher the efficiency.

### 7.2. Experimental Results

#### 7.2.1. BP Model Assessment

In the experiments, the data set is divided into two subsets, 67% for training and 33% for testing. MSE and $R^{2}$ are adopted to calculate the residuals for each water parameter. Table 5 lists the training results and test results for six water quality parameters with BP models. As shown in Table 5, the computed *MSE*s of each water parameter are all below 0.035, which indicates that the training subset does not have much noise data and can reflect the actual water quality conditions. Moreover, the difference between the measured and estimated value for each parameter is quite small in the test phase. The corresponding $R^{2}$ values of each parameter are acceptable. Therefore, it can be seen that the BP neural network model can be used to identify water quality state “normal” or “outlier”.

#### 7.2.2. Accuracy Analysis

**Figure 9:**
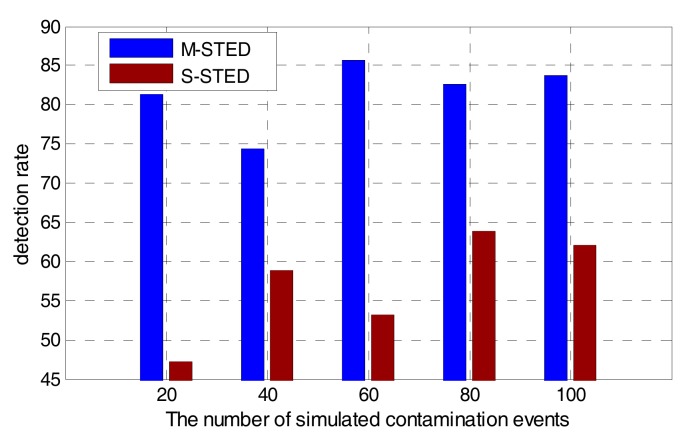
Comparison of detection rates between M-STED and S-STED.

**Figure 10:**
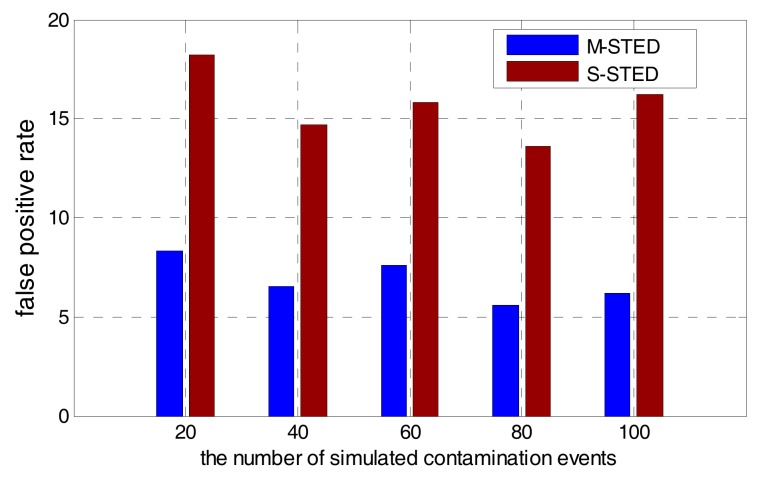
Comparison of false alarm rates between M-STED and S-STED.

To improve the accuracy of event detection, M-STED adopts BP models to establish the prediction model for each water quality parameter. Different water quality parameters have different predictive abilities for contamination events; it should allocate the appropriate weights to make a unified decision. Contamination event detection based on the multivariate parameters can greatly reduce the false alarms and improve the detection rate, compared with the detection based on the univariate parameter. As shown in Figure 9 and Figure 10, when the number of simulated contamination events vary from 20 to 100, the contamination event detection rate falls to a range between 45–65% with the S-STED method, while the detection rate is greater than 75% with the M-STED method. The average detection rate is more than 80% with M-STED. The detection rate of M-STED is 40% higher than that of S-STED. Meanwhile, the false alarm rates of M-STED and S-STED are lower than 9% and greater than 14%, respectively. Obviously, M-STED can effectively reduce the false alarm rates.

**Figure 11:**
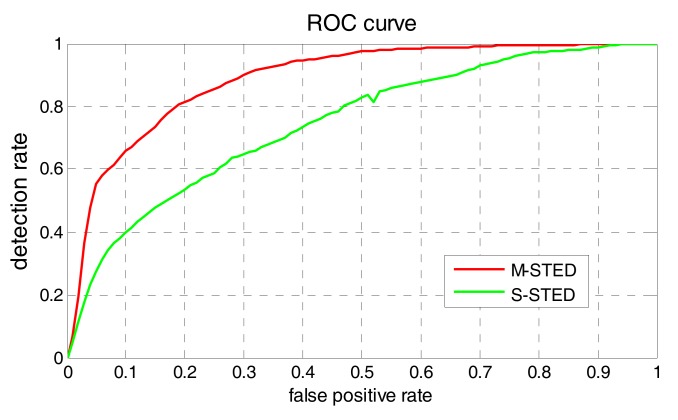
Comparison of ROC (receiver operating curve) between M-STED and S-STED.

In statistics, a ROC (receiver operating curve) is created by plotting the true positive rate against the false positive rate at various threshold settings. In our experiments, the true positive rate is denoted as RD, which represents the ratio of the number of the detected contamination events to the number of the actual contamination events. The false positive rate is denoted as FAR, which is the ratio of the number of the detected false alarms to the total number of the determined contamination events. In general, if the probability distributions for both detection and false alarm are known, the ROC curve can be generated by plotting the cumulative distribution function (area under the probability distribution) of detection rate in the y-axis versus the cumulative distribution function of the false alarm rate in the x-axis. Therefore, RD and FAR can be used to construct ROC, which visually depict the same information as the confusion matrix demonstrating the fundamental performance trade-off between RD and FAR in a much more intuitive way. Figure 11 illustrates the ROC of S-STED and M-STED, respectively. As shown in Figure 11, area under ROC of M-STED is significantly greater than that of S-STED.

Based on the experimental results analysis, M-STED has better performance in terms of the detection rate and false alarm rate than S-STED, which relies on a single water parameter.

#### 7.2.3. Detection Delay

**Figure 12:**
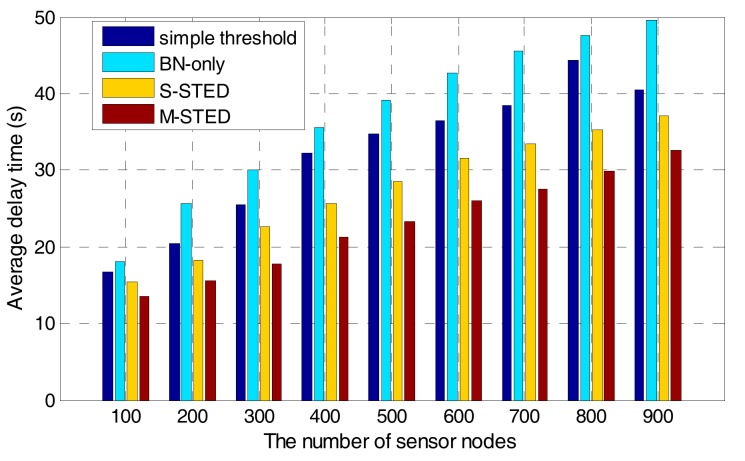
The average delay time in the different node densities.

To evaluate the detection delay of different methods, different numbers of sensors are selected in the same area to execute the proposed M-STED, S-STED, BN-only, and simple threshold approaches. As shown in Figure 12, with the increase of the number of sensor nodes, the average delay of the four approaches significantly increase. The average delay time of the proposed M-STED is smaller than that of S-STED, BN-only, and the simple threshold one. The results show that the communication overhead increases with the increase of the number of nodes. However, the proposed M-STED approach exhibits a slower increase than the other three approaches. The reason is that Rule **K** algorithm is adopted to select part of the nodes as backbone nodes for forwarding the sensed data, which can greatly reduce the communication overhead. Therefore, the proposed M-STED can achieve shorter average delay for contamination event detection. While BN-only and simple threshold approaches need to collect all of the sensed data, resulting in a large communication overhead. The response time of event detection is longer than that of the proposed M-STED approach.

#### 7.2.4. Scalability

**Figure 13:**
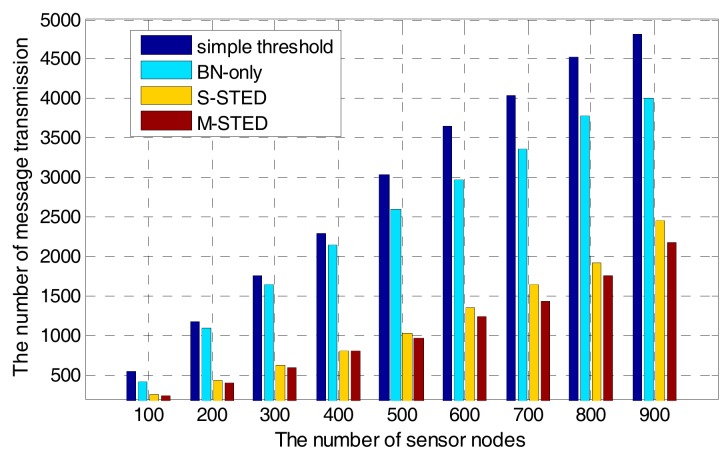
Scalability with an increased number of nodes.

The communication complexity of the proposed M-STED approach depends on the local transition of the sensed values. If the nodes are not in the CDS, detecting temporal events requires no communication overhead, because the nodes run the algorithm individually. Whenever a temporal event is detected by the backbone nodes, they broadcast the corresponding messages and collect the sensed data. If the nodes in the CDS are in the “outlier” state, the communication overhead and the message transmission increase when the number of sensor nodes increases. From Figure 13, it can be seen that with the increase of the number of sensor nodes, the number of message transmission also increases for all of the event detection methods. It is obvious that the increment speeds of the S-STED and M-STED with Rule **K** algorithm are slower than those of BN-only and simple threshold approaches. The S-STED and M-STED approaches can achieve better performance on data transmission. The reason is that Rule **K** algorithm can optimally reduce the number of sensor nodes, resulting in a low communication overhead. On the contrary, without an optimal node selection procedure, BN-only and simple threshold approaches exhibit a linear increase of communication overhead when the number of sensor nodes increases.

Furthermore, the scale of water supply networks also causes side effect on the efficiency of performance. As the number of sensor nodes increases, the average delay time of the proposed M-STED approach also grows. However, the increase speed is slower than that of BN-only and simple threshold approaches. The weak linear correlation of the number of sensor nodes and communication overhead shows good performance on scalability for the proposed M-STED approach.

## 8. Conclusions

To improve the accuracy of contamination event detection in the agricultural water supply networks, a spatial–temporal-based event detection approach with multivariate time-series data for water quality monitoring (M-STED) was proposed. M-STED adopts Rule **K** algorithm to select backbone nodes, which collect water quality parameters to reduce the transmitted data. Then, the BP neural networks models and sequential Bayesian analysis are adopted to detect the “outlier” nodes. Finally, a spatial model is established with BN to estimate and trace the state of “outlier” nodes to determine a contamination event. The experimental results indicate that the average detection rate is more than 80% with M-STED and the false detection rate is lower than 9%, respectively. The M-STED algorithm can improve the rate of detection by about 40% and reduce the false alarm rate by about 45%, compared with the S-STED algorithm. Moreover, the proposed M-STED can exhibit better performance in terms of detection delay and scalability.

## Figures and Tables

**Table 1:** Notations of four conditions.

Estimated Conditions	Actual Normal Operation	Actual Outlier Operation
Normal operation	True Negative (TN)	False Negative (FN)
Outlier operation	False Positive (FP)	True Positive (TP)

**Table 2:** Updated probability of contamination events in a single node.

Time	Mon 08:00	Mon 08:20	Mon 08:40	Mon 09:00	Mon 16:00	Mon 16:20	Mon 16:40	Mon 17:00
**true state**	FALSE	TRUE	TRUE	TRUE	TRUE	TRUE	TRUE	TRUE
free chlorine [mg/L]	4.19 × 10^−5^	4.05 × 10^−1^	*9.50 × 10^−1^*	*9.50 × 10^−1^*	*8.69 × 10^−2^*	4.18 × 10^−2^	3.50 × 10^−1^	1.52 × 10^−1^
EC [mS/cm]	1.00 × 10^−5^	1.00 × 10^−5^	1.00 × 10^−5^	1.00 × 10^−5^	2.59 × 10^−1^	*8.01 × 10^−1^*	3.02 × 10^−1^	2.16 × 10^−2^
pH	1.00 × 10^−5^	1.00 × 10^−5^	1.00 × 10^−5^	1.00 × 10^−5^	1.00 × 10^−5^	1.00 × 10^−5^	1.00 × 10^−5^	1.00 × 10^−5^
Temperature	1.00 × 10^−5^	1.00 × 10^−5^	1.00 × 10^−5^	1.00 × 10^−5^	1.00 × 10^−5^	1.00 × 10^−5^	1.00 × 10^−5^	1.00 × 10^−5^
TOC [ppb]	1.87 × 10^−5^	*9.46 × 10^−1^*	*9.50 × 10^−1^*	*9.50 × 10^−1^*	*9.50 × 10^−1^*	*9.50 × 10^−1^*	2.01 × 10^−1^	4.02 × 10^−1^
Turbidity [NTU]	1.00 × 10^−5^	1.00 × 10^−5^	5.34 × 10^−2^	*9.50 × 10^−1^*	*9.50 × 10^−1^*	*9.50 × 10^−1^*	*9.50 × 10^−1^*	4.02 × 10^−1^
Alarm (1)	FALSE	FALSE	**TRUE**	**TRUE**	**TRUE**	FALSE	FALSE	FALSE
Alarm (2)	FALSE	FALSE	FALSE	FALSE	FALSE	**TRUE**	FALSE	FALSE
Alarm (3)	FALSE	FALSE	FALSE	FALSE	FALSE	FALSE	FALSE	FALSE
Alarm (4)	FALSE	FALSE	FALSE	FALSE	FALSE	FALSE	FALSE	FALSE
Alarm (5)	FALSE	**TRUE**	**TRUE**	**TRUE**	**TRUE**	**TRUE**	FALSE	FALSE
Alarm (6)	FALSE	FALSE	FALSE	**TRUE**	**TRUE**	**TRUE**	**TRUE**	FALSE

Notes: the underline italic numbers denote that the values are greater than the threshold. The bold texts mean that the results of alarms are true.

**Table 3:** The temporal event probability table of sensor $S_{1}$

State	$P(S_{1})$
$state_{1}$	0.2
$state_{2}$	0.8

**Table 4:** The conditional probability table of sensor $S_{2}$ and $S_{3}$.

$S_{1}$	$S_{2}$	$P(S_{2})$	$S_{1}$	$S_{3}$	$P(S_{3})$
$state_{1}$	$state_{1}$	0.8	$state_{1}$	$state_{1}$	0.8
$state_{1}$	$state_{2}$	0.4	$state_{1}$	$state_{2}$	0.3
$state_{2}$	$state_{1}$	0.3	$state_{2}$	$state_{1}$	0.2
$state_{2}$	$state_{2}$	0.5	$state_{2}$	$state_{2}$	0.6

**Table 5:** Training results and test results with BP model.

Phase	Parameters		Free Chlorine [mg/L]	EC mS/cm	pH	Temperature [°C]	TOC [ppb]	Turbidity [NTU]
Training phase	$mean_{1}$	measured value	1.945	77.670	9.039	17.253	0.966	0.220
		estimated value	1.947	78.320	9.040	17.253	0.960	0.223
	$mean_{2}$	measured value	1.945	77.670	9.039	17.253	0.966	0.220
		estimated value	1.947	78.32	9.04	17.253	0.960	0.223
	$R^{2}$		0.923	0.986	0.999	0.999	0.685	0.639
	MSE		0.007	0.0347	0.000	0.005	0.076	0.008
Test phase	$mean_{1}$	measured value	2.002	87.207	9.159	18.078	1.031	0.198
		estimated value	2.010	88.359	9.151	18.086	1.021	0.225
	$mean_{2}$	measured value	0.145	55.804	0.240	1.302	1.214	1.351
		estimated value	0.075	52.244	0.215	1.189	1.452	1.439
	$R^{2}$		0.625	0.914	0.690	0.779	0.654	0.654
	MSE		0.016	0.502	0.018	0.377	0.732	1.379
